# When a Large Left Hemisphere Stroke is All Right for Language, Praxis, and Visual Attention: A Case Report

**DOI:** 10.1212/wn9.0000000000000053

**Published:** 2025-12

**Authors:** Elizabeth H.T. Chang, Peter E. Turkeltaub, Anna Seydell-Greenwald

**Affiliations:** 1 Center for Brain Plasticity and Recovery, Georgetown University Medical Center; 2 Research Division, MedStar National Rehabilitation Hospital.

## Abstract

**Objectives:**

To describe a rare case of large left hemisphere (LH) middle cerebral artery (MCA) stroke with intact language, praxis, visuospatial cognition, and attention, suggesting lateralization of all these functions to the intact right hemisphere (RH).

**Methods:**

A 53-year-old right-handed woman who had a stroke at age 42 was enrolled in a study of long-term stroke outcomes. She underwent standardized behavioral assessments and functional MRI (fMRI) with a visual field test and an adaptive semantic matching task.

**Results:**

Despite a 432 cm^3^ area of encephalomalacia, encompassing both MCA and posterior anterior cerebral artery territories of the LH, she exhibited intact language both acutely and chronically (Western Aphasia Battery Aphasia Quotient = 99.2) and normal praxis. fMRI demonstrated right-hemisphere dominance for language. Visuospatial cognition and attention were preserved (WASI II-Block Design; Behavioral Inattention Test-conventional subtests) despite right hemianopia.

**Discussion:**

This case suggests that language, praxis, and visuospatial functions can simultaneously lateralize to the RH. Although previous cases report reciprocal hemispheric reorganization, such as in crossed aphasia, this case of “crossed non-aphasia” supports the view that lateralization of functions can occur independently. Clinically, this rare pattern of atypical lateralization should be considered when expected poststroke deficits are absent.

## Introduction

Left hemisphere (LH) strokes commonly result in language impairments, while right hemisphere (RH) strokes are associated with visuospatial and attentional deficits. Here, we present a case of a woman who had a large LH middle cerebral artery (MCA) ischemic stroke at age 42 and exhibits neither aphasia nor the typical cognitive sequelae of RH stroke. This case highlights a rare instance of simultaneous RH dominance for major cognitive functions typically lateralized to opposite hemispheres.

## Methods

### Experimental Design

The patient enrolled in two IRB-approved observational studies of chronic stroke outcomes at Georgetown University. Clinical history, including acute presentation, imaging, and past medical history, was obtained through electronic medical records. Standardized behavioral assessments were conducted at 61- and 86-month poststroke. The test battery included Montreal Cognitive Assessment (MoCA), Western Aphasia Battery (WAB), Apraxia Battery for adults, Wechsler Abbreviated Scale of Intelligence-II (WASI-II), Behavioral Inattention Test-conventional subtests (BIT-c), and oral real and pseudoword reading and spelling tasks. Language outcomes were compared with those of 95 LH stroke survivors. Reading and spelling performance was compared with those of 17 education-matched healthy controls. Visuospatial outcomes were compared with those of 32 RH stroke survivors. Demographic information on all three cohorts is summarized in eTable 1. Both studies enrolled adult stroke survivors without other major neurologic conditions >6-months poststroke, as well as demographically matched neurologically healthy adults.

To examine neural correlates of language, we used a well-validated fMRI adaptive semantic matching paradigm^1^ in which participants see pairs of written words and decide whether the words are semantically related, contrasted with a control condition during which participants judge visual similarity between pairs of nonlinguistic symbol strings. Visual field mapping was performed using a flickering checkerboard stimulus.

MRI was acquired on a 3T Siemens Prisma scanner. High-resolution T1-weighted MPRAGE and FLAIR sequences were obtained to visualize anatomy and assist in lesion tracing. Lesions were manually segmented on coregistered MPRAGE and FLAIR images using ITK-SNAP imaging and segmentation software by a board-certified neurologist (P.E.T.). Images and lesion masks were normalized to MNI space using the Clinical Toolbox Older Adult Template.^2^

### Standard Protocol Approvals, Registrations, and Patient Consents

The participant provided written informed consent under Georgetown University IRB-approved protocols: IRB2017–0305 and IRB2013–0964. She also reviewed the manuscript and signed a consent-to-disclose form. To protect confidentiality, a randomly generated three-letter code (ECP) is used instead of patient initials, and data not published within this article are not shared.

## Results

**Figure 1:**
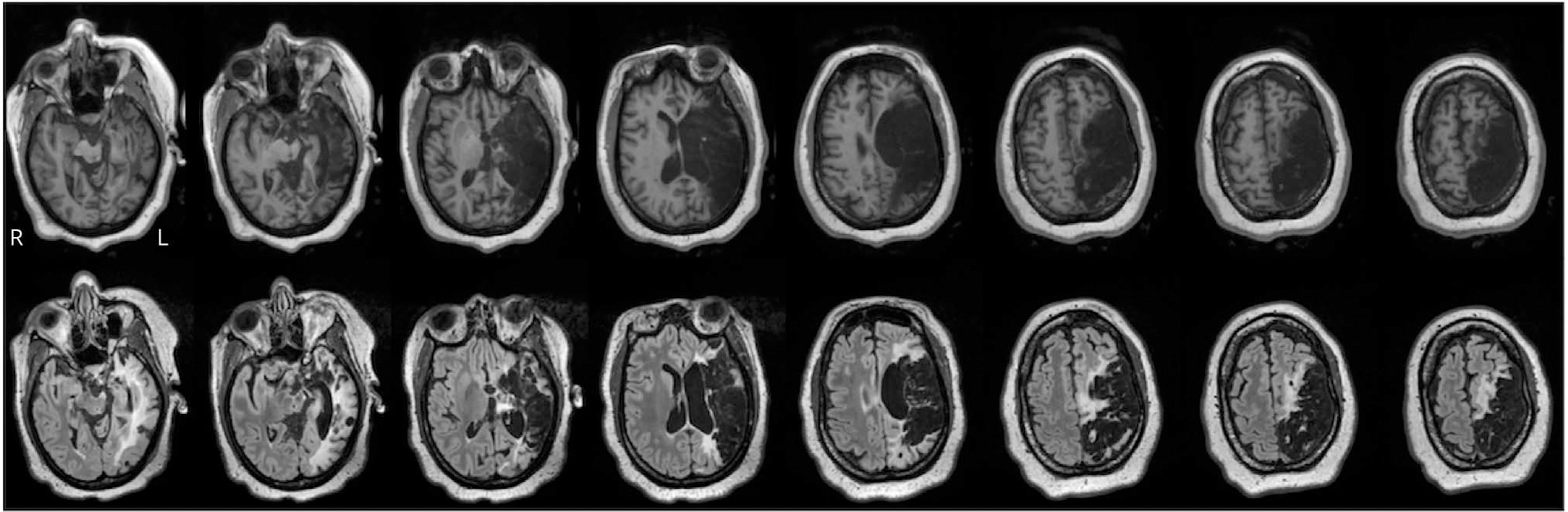
MRI Acquired 61-Month Poststroke Flair and T1-weighted MRI acquired 61-month poststroke showing left hemisphere stroke encompassing MCA and posterior ACA territories. ACA = anterior cerebral artery; MCA = middle cerebral artery.

ECP was a 42-year-old right-handed high school graduate who presented with right-sided hemiparesis, partial left gaze deviation, dysarthria, and altered mental state following a motor vehicle accident. She was found to have an occlusion of the left proximal internal carotid artery causing acute left middle and anterior cerebral artery (ACA) territory ischemia with a significant mass effect. ECP was ineligible for tissue plasminogen activator treatment or mechanical thrombectomy due to last seen normal being well outside the therapeutic window. She was instead treated with mannitol to manage cerebral edema. The NIH Stroke Scale (NIHSS) was 13 on admission. She was enrolled in an observational study of chronic stroke outcomes 61 months after the stroke. At this time, her NIHSS was 10 for right hemianopsia, facial droop, hemiparesis, hemisensory loss, and tactile extinction. Imaging revealed substantial area of encephalomalacia of 432 cm^3^ affecting left frontal, temporal, and parietal regions encompassing the entire MCA territory and the posterior portion of the ACA territory (Figure 1).

**Figure 2:**
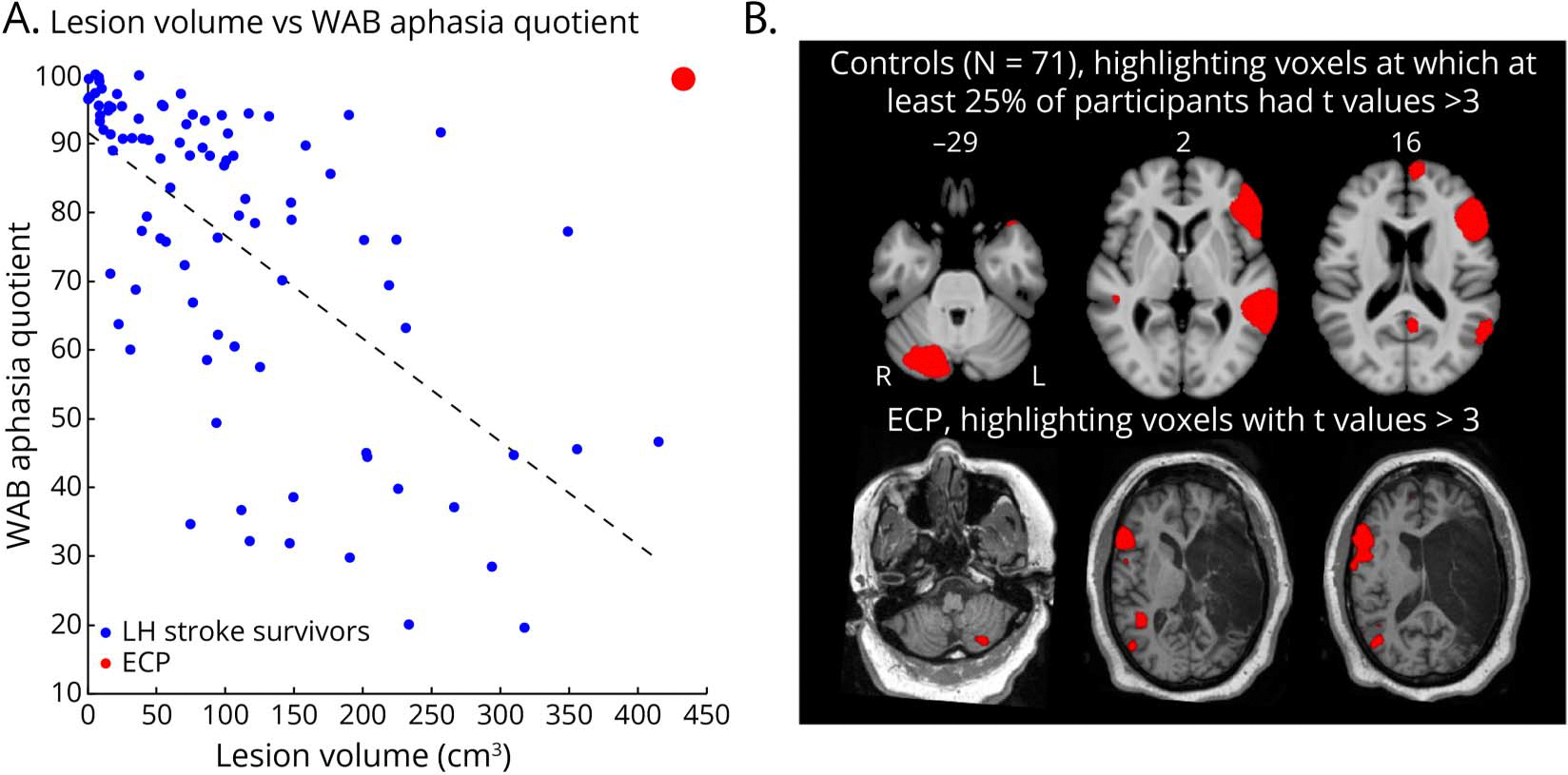
Relationship Between Lesion Volume and WAB and fMRI Activation (A) Scatterplot of lesion volume (cm^3^) vs Western Aphasia Battery Aphasia Quotient (WAB AQ), illustrating inverse relationship between lesion size and language (dashed line) across LH stroke survivors (blue). ECP, highlighted in red, demonstrates preserved language function despite having the largest lesion volume in the cohort. (B) fMRI activation on an adaptive semantic matching task shows robust activity in areas commonly associated with language processing, including the left inferior temporal gyrus, left posterior superior temporal cortex, and right cerebellum in 71 typical older controls (map shows voxels active in >25% of participants at a single-voxel threshold of *t* > 3). In a mirror-reversal of this typical pattern, ECP demonstrates activation in homotopic regions of the right cerebral cortex, with left cerebellar activation (map shown at single-voxel threshold of *t* > 3). To correct for multiple comparisons, we applied cluster-size thresholds to each individual map that control the family-wise error rate. fMRI = functional MRI.

Despite the lesion’s size and considerable overlap with tissue typically associated with language function,^3–5^ ECP displayed no language deficits in the acute phase based on both medical records and retrospective history provided by ECP and family members. Language assessments at 61- and 86-month post-stroke also revealed no aphasia. On the WAB,^6^ where a score greater than 93.8 indicates no aphasia and a score of 100 indicates perfect performance, ECP scored 99.2. Among a cohort of 95 LH stroke survivors who participated in the same study, the median score was 87.5. All other participants with scores above 93.8 had lesions less than half the size of that of ECP (Figure 2A). ECP performed within 1 SD of the mean of an education-matched healthy control group on reading and spelling tasks (real word reading: z = 0.26; pseudoword reading: z = 0.15; typing to dictation: z = −0.66). Limb and oral praxis, which are typically left-lateralized,^7^ were also intact based on the Apraxia Battery for Adults^8^; Limb 50/50, Oral 48/50.

On the adaptive semantic matching fMRI paradigm,^1^ where neurologically healthy adults showed the expected pattern of activation in the left inferior gyrus (IFG), temporal regions, and right cerebellum (Figure 2B), ECP demonstrated robust activation in the right IFG and right temporal regions, along with the left cerebellum, showing a mirror reversal of the typical activation pattern (Figure 2B). Premorbidly, ECP was right-handed (retrospective Edinburgh Handedness Inventory Index = 70),^9^ and she denied having left-handed first-degree relatives. She also denied any history of symptoms indicative of left-hemisphere injury or dysfunction early in life that could have explained her atypical language dominance.

**Figure 3:**
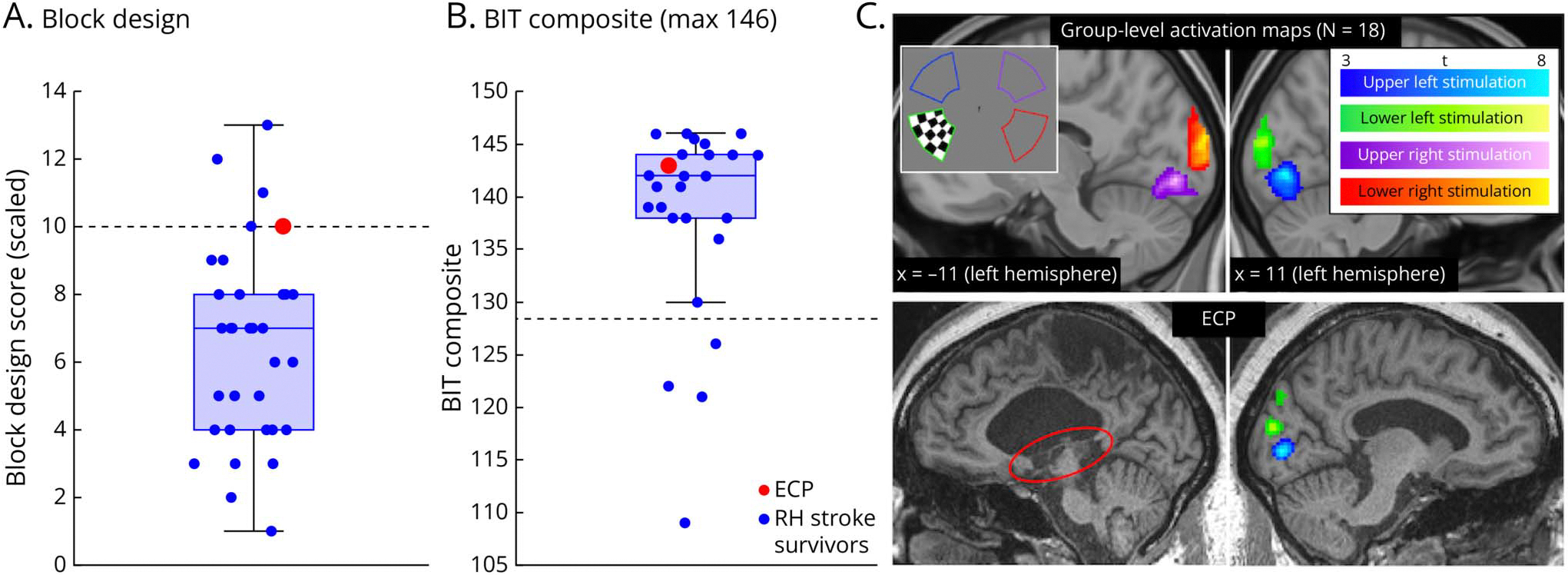
Visuospatial Task Performance and Visual Field Activation Maps (A) ECP’s Scaled Block Design score of 10 (red) compared with 31 RH stroke survivors (blue, median = 7), with a population mean of 10 ( ± 3) (dashed line). (B) ECP’s Behavioral Inattention Test (BIT) composite score of 143 (red) falls clearly above the cutoff (dashed line), demonstrating no evidence of hemispatial neglect. (C, top) fMRI of visual field test showing robust left occipital activations in response to right visual field stimuli and right occipital activations in response to left visual field stimuli in 18 healthy controls. Maps are thresholded at *t* > 3 and display only the largest activation cluster for stimulation in the color-coded quadrant. (C, bottom) ECP’s activation maps (also thresholded at *t* > 3 in combination with cluster-size threshold to control the family-wise error rate) shows similarly robust response in the right hemisphere but no significant activation in the left hemisphere in response to right visual field stimuli, suggesting a right field cut. The sagittal view of the left hemisphere also reveals damage to the visual pathway (red oval). fMRI = functional MRI; RH = right hemisphere.

Further testing at 86-month poststroke revealed no visuospatial or attentional deficits. On the WASI-II Block Design,^10^ ECP’s scaled score of 10 corresponded to the healthy norming sample and exceeded the mean of 32 other RH stroke survivors enrolled in the same study (Figure 3A). ECP scored 29/30 on the MoCA, with a 3/3 on the serial sevens section. On the Behavioral Inattention Test conventional subset (BIT-c),^11^ she scored 143/146, with no lateralized deficits on cancellation, line bisection, or drawing tasks (Figure 3B). A computerized line bisection task with reversed motor mapping^12^ also revealed no motor-intentional neglect.

Visual field testing confirmed a right homonymous hemianopsia. fMRI showed absent activation in the left occipital cortex in response to right visual field stimulation (Figure 3C), and structural imaging revealed damage to the left optic radiation and lateral geniculate nucleus. Given this combination of right hemianopsia and lack of lateralized deficits on neglect tests with free visual exploration, the presence of tactile extinction on the NIHSS likely reflects partial hemisensory loss or tactile inattention without visuospatial neglect.

## Discussion

This case presents a rare instance of preserved cognitive performance for language, praxis, and visuospatial functions following a large left MCA ischemic stroke. The absence of aphasia and the presence of right-lateralized language activation suggest premorbid RH dominance for language in this right-handed individual. Although this conclusion is inferential, it is consistent with epidemiologic evidence that a minority of right-handed individuals (~7.5%) demonstrate right-hemisphere language.^13^

Most published examples of atypical lateralization describe crossed aphasia after right-hemisphere stroke in right-handed individuals (eTable 2). Selnes et al.^14^ reported a case in which a left MCA infarct spared language but produced neglect and other deficits, typically associated with right-hemisphere injury. This is consistent with neuroimaging work showing that atypical lateralization of the RH is often accompanied by lateralization of typically right-lateralized functions (such as visuospatial processing) to the left, maintain a functional hemispheric segregation.^15^ In violation of this mirror pattern, ECP preserved both language and right-hemisphere functions. These findings support the view that lateralization biases for different cognitive domains can vary independently, allowing, in rare instances, lateralization of language, praxis, and visuospatial functions to the same hemisphere.

Clinically, atypical lateralization is often noted based on crossed deficits (e.g., aphasia resulting from RH stroke). This case demonstrates that alternative patterns of hemispheric organization may also occur and should be considered during assessment and treatment planning when anticipated cognitive deficits are absent.

## Supplementary Material

eTables1and2
