# Supplementary material for: When a Large Left Hemisphere Stroke is All Right for Language, Praxis, and Visual Attention: A Case Report
Source: Neurol Open Access. Author manuscript; Available in PMC 2026 Jan 22. (PMC12823144; doi:10.1212/wn9.0000000000000053)
Supplement: eTables1and2 [file NIHMS2127260-supplement-eTables1and2.pdf]

	**Left Hemisphere Stroke (n=95)**	**Right Hemisphere Stroke (n=31)**	**Education-Matched Controls (n=17)**
**Age** (Mean (SD))	60.0 (11.9)	59.5 (11.2)	63 (12)
**Sex**	53 M/ 42 F	18 M/ 13 F	9 F/ 8 M
**Race**	39 Black or African American, 55 White or Caucasian, 1 More than One Race	16 Black or African American,13 White or Caucasian, 2 Asian	12 Black or African American, 5 White or Caucasian

eTable 1: Table of demographic for comparison groups. Language tasks scores were compared to those of LH stroke survivors; visuospatial tasks were compared to those of RH stroke survivors. Reading and spelling tasks were compared to an education-matched group of healthy controls.

Study	Patient	Handedness	Stroke Location	Aphasia	Praxis	Visuospatial/ Attention Deficit
(Brown & Wilson, 1973)	42F	dextral	Right Frontoparietal Infarct	+	-	-
(Alexander et al., 1989) Case 1	54M	dextral	Right hemisphere vascular lesion	+	-	-
(Alexander et al., 1989) Case 2	65F	dextral	Right supramarginal + left frontal lesion	+	+	+
(Sheehy & Haines, 2004)	67F	dextral	Right MCA infarct	+	n/a	+
(Selnes et al., 1991)	81M	dextral	Left MCA infarct	-	+	+
ECP	42F	dextral	Left MCA infarct	-	-	-

eTable 2. Published case reports of atypical lateralization compared with the present case, highlighted in blue. Reported cognitive functions include language, praxis, and visuospatial/attentional function. "+" indicates impairment in that domain, "-" indicates intact performance, and "n/a" indicates the domain was not reported. Notably, this table highlights that while most prior reports describe crossed aphasia after right hemisphere stroke, ECP represents a rare instance of "crossed non-aphasia" following a large left MCA infarct.

References for eTables:

Alexander, M. P., Fischette, M. R., & Fischer, R. S. (1989). CROSSED APHASIAS CAN BE MIRROR IMAGE OR ANOMALOUS: CASE REPORTS, REVIEW AND HYPOTHESIS. *Brain*, *112*(4), 953–973. https://doi.org/10.1093/brain/112.4.953

Brown, J. W., & Wilson, F. R. (1973). Crossed aphasia in a dextral: A case report. *Neurology*, *23*(9), 907–907. https://doi.org/10.1212/WNL.23.9.907

Selnes, O. A., Pestronk, A., Hart, J., & Gordon, B. (1991). Limb apraxia without aphasia from a left sided lesion in a right handed patient. *Journal of Neurology, Neurosurgery & Psychiatry*, *54*(8), 734–737. https://doi.org/10.1136/jnnp.54.8.734

Sheehy, L. M., & Haines, M. E. (2004). Crossed Wernicke's aphasia: A case report. *Brain and Language*, *89*(1), 203–206. https://doi.org/10.1016/S0093-934X(03)00365-1
